# A Multilevel Analysis of the Association Between Quality of Antenatal Care and Folic Acid Supplementation During Pregnancy Among Guatemalan Women

**DOI:** 10.1155/ghe3/4427791

**Published:** 2025-06-12

**Authors:** Sueny P. Lima dos Santos, Raegan Yuncker, Ilana R. A. Chertok, Zelalem T. Haile

**Affiliations:** ^1^Graduate College, Ohio University, Athens, Ohio, USA; ^2^Center for Nutrition and Health Impact, Omaha, Nebraska, USA; ^3^Global Health Initiative, College of Health Sciences and Professions, Grover Center, Ohio University, Athens, Ohio, USA; ^4^School of Nursing, College of Health Sciences and Professions, Ohio University, Athens, Ohio, USA; ^5^Department of Social Medicine, Ohio University Heritage College of Osteopathic Medicine, Dublin, Ohio, USA; ^6^Global Health Initiative, College of Health Sciences and Professions, Athens, Ohio, USA

**Keywords:** antenatal care, DHS, folic acid supplementation, guatemala, maternal and child health

## Abstract

**Background:** Folic acid supplementation during pregnancy is essential for preventing neural tube defects and other congenital anomalies. Despite global recommendations, supplementation remains suboptimal in many low- and middle-income countries, including Guatemala, where disparities persist across regions and populations.

**Objective:** To investigate the association between the quality of antenatal care and folic acid supplementation among pregnant women in Guatemala.

**Design and Setting:** This cross-sectional study used data from the 2014-2015 Encuesta Nacional de Salud Materno Infantil (ENSMI), part of the Demographic and Health Survey (DHS). A total of 9523 women aged 15–49 with children under two years were included. Folic acid supplementation was assessed through self-reported responses to survey questions. Multilevel logistic regression examined the association between antenatal care quality and folic acid supplementation, accounting for individual, household, and community-level factors.

**Results:** Overall, 15.4% of women reported not taking folic acid during pregnancy. Lower folic acid supplementation was most notable among women who received no or inadequate antenatal care, indigenous women, and those living in socioeconomically disadvantaged communities. Women without antenatal care had 97% lower odds of folic acid supplementation compared with those with adequate care (OR = 0.03, 95% CI: 0.02–0.04, and *p* < 0.001), while intermediate care was associated with 41% lower odds (OR = 0.59, 95% CI: 0.47–0.74, and *p* < 0.001). Indigenous women had 26% lower odds of supplementation (OR = 0.74, 95% CI: 0.63–0.86, and *p* < 0.001), and women in communities with high levels of no media exposure had 33% lower odds of folic acid supplementation (OR = 0.67 and 95% CI: 0.53–0.84).

**Conclusions:** Quality antenatal care plays a critical role in improving maternal nutrition behaviors. These findings underscore the need for targeted interventions, such as culturally tailored education, mass media campaigns, and improved access to antenatal careto increase folic acid supplementation among pregnant women in Guatemala.

## 1. Introduction

The role of folic acid supplementation before and during the early stages of pregnancy in mitigating the risk of neural tube defects is well established. However, the global disparities, especially in low- and middle-income countries (LMICs), highlight the pressing need for structured research methodologies and extensive data-driven interventions [1]. This is particularly true in Guatemala, where the most recent and comprehensive data comes from the 2014-2015 Demographic and Health Survey (DHS). These data remain critically important despite its age as subsequent DHS cycles, including the 2022 survey, were unfortunately canceled.

Guidelines from organizations like the World Health Organization (WHO) suggest a daily dose of 400 μg of folic acid for women of childbearing age [2]. While these guidelines set a universal standard, real-world implementation varies significantly due to supplement availability, affordability, and cultural dietary practices. In Guatemala, the assumption of uniform dietary patterns needs to be revised, necessitating tailored strategies that accommodate diverse cultural settings. Previous local studies have explored innovative approaches, such as fortifying commonly used items such as iodized salt and vitamin A-fortified sugar, demonstrating the potential of context-specific interventions [3, 4].

Despite long-standing folic acid fortification policies in Guatemala, approximately 50% of women of reproductive age (WRA) are folate insufficient, with regional variations ranging from 18% to 81% [5]. Socioeconomic disparities significantly influence folate status, with indigenous, rural, and low-income populations experiencing the highest rates of deficiency due to limited access to fortified foods [6]. Based on red blood cell folate concentrations, the estimated national neural tube defect (NTD) risk is 1.4 per 1000 live births, varying regionally from 1.1 to 2.6 per 1000 live births [5].

Given the varied dietary habits and economic resources across different regions [7], healthcare providers in Guatemala must have access to comprehensive and current data to educate women about the importance of folic acid. Continuous and updated data collection by the DHS is crucial for understanding the cyclical trends in health practices and outcomes. Such data facilitate the identification of progress and gaps in antenatal care practices and enables the development of responsive and informed health policies and programs in LMICs [8].

Given these disparities, the role of antenatal care becomes even more critical in advancing maternal and child health outcomes, as it shows a key role in reducing maternal mortality, improving pregnancy outcomes, and ensuring early interventions for fetal health [9, 10]. Research highlights that maternal health literacy is a critical determinant in accessing and utilizing antenatal care services, particularly among low-income women [10], reinforcing the need for targeted educational and healthcare interventions. However, the quality of these services can vary across different settings due to factors such as healthcare infrastructure, resource availability, healthcare-provider training, and patient-provider interactions [11, 12]. Similar disparities have been observed in fragile healthcare systems, where access to basic maternal services is compromised by financial constraints, infrastructure deficiencies, and workforce shortages [13].

Limited research exists on the link between the quality of ANC and folic acid supplementation in Guatemala, particularly among vulnerable populations, such as rural, low-income, and indigenous women who face significant barriers to accessing healthcare and fortified foods. Although the 2014-2015 DHS data for Guatemala are nearly a decade old, it remains the most recent population-based dataset that captured information on folic acid supplementation. By examining the association between the quality of antenatal care and folic acid supplementation among pregnant women in Guatemala, this study offers evidence that can inform targeted maternal health interventions.

## 2. Methods

### 2.1. Design

This analysis utilized nationally representative cross-sectional data from the 2014-2015 Guatemala Encuesta Nacional de Salud Materno Infantil (ENSMI), part of the DHS. A detailed description of the 2014-2015 Guatemala ENSMI design and methods is available elsewhere [14]. In brief, the survey employed a multistage stratified sampling technique that selected 36 randomly selected sectors from Guatemala's 22 departments with a proportional distribution of urban and rural areas. Approximately 26 households were selected per sector, resulting in 21,383 surveyed households. In this set of interviewed households, 26,767 women aged 15–49 participated in the survey, and 12,440 had given birth within the last five years. The current study is restricted to women who had given birth within the last 5 years and responded to questions related to the pregnancy of the most recent birth (*N* = 9542) following the standard DHS methodology, which collects detailed pregnancy and birth-related data only for births occurring within this timeframe. We further excluded women with missing data on folic acid supplementation (*n* = 3), number of antenatal care contacts *n* = 5), the timing of the first antenatal care visit (*n* = 2), components of antenatal care (*n* = 3), and other covariates adjusted in the multivariable model (*n* = 6). The final sample size consisted of 9523 women. Data were collected by the Ministerio de Salud Pública, Asistencia Social (MSPAS), with technical assistance from the DHS program Ministerio de Salud Pública y Asistencia Social [14].

### 2.2. Setting

Guatemala is located in Central America, bordered by Mexico to the northwest, Belize to the east, Honduras to the southeast, El Salvador to the south, and the North Pacific Ocean to the southwest. According to national agencies, in 2014, Guatemala had an estimated 15,608,000 people, with approximately 55.2% residing in urban areas.

### 2.3. Measurement

The outcome of interest was folic acid supplementation during pregnancy, which was determined based on women's responses to the question, “During this pregnancy, did you take some pills of folic acid?” Responses were coded as yes or no. The main independent variable was the quality of antenatal care. We operationalized the quality of antenatal care utilizing an indicator informed by Lima dos Santos et al. [15], which considers maternal reports of their antenatal care experiences. This measure includes the total number of antenatal care contacts attended, the timing of the first antenatal visit, and receipt of four key components: blood pressure measurements, blood samples, urine samples, and weight measured. We categorized antenatal care quality into three levels: ‘adequate' defined as a minimum of four contacts initiated within the first trimester along with all health checks; ‘intermediate' for less frequent contacts, later initiation, or incomplete health checks; and ‘none' for no reported antenatal care. This operationalization partially aligns with the WHO recommendations for positive pregnancy experience, which encourage early initiation and provision of key clinical assessments, but it was adapted based on the components of ANC available in the 2014-2015 Guatemala DHS, which did not capture all WHO-recommended interventions.

Potential confounder variables were identified from the literature search [15–22]. These include maternal age (15–19, 20–24, 25–29, 30–34, and 35–49), maternal education (none, primary, secondary, or above), marital status (never married, married, or living together), household wealth index (poor, middle, and rich), maternal employment status (unemployed and employed), place of residence (urban and rural), number of children (1, 2–4, 5, or more), pregnancy intention of index child (wanted then, wanted later, and did not want), auto identified as indigenous or not, and access to mass media sources based on participants' responses to how often they read a newspaper, listened to the radio, or watched television. Those who responded that they utilized one of these sources at least once a week were exposed to mass media. The sum of these responses was then created to determine the number of sources of mass media categories (none, one, two, or three mass media sources).

### 2.4. Ethical Considerations

Before collecting data, DHS program workers obtained informed consent from participants and assured confidentiality. The DHS program approved access to and use of the data before the secondary data analysis. The researchers' academic institutional review board determined the study to be exempt due to the unidentifiable participants and anonymous data.

### 2.5. Statistical Analysis

Frequencies and proportions were used to describe the characteristics of the study sample. The Rao–Scott chi-square test was used to examine differences in folic acid supplementation by the characteristics of the study sample.

We employed the SURVEYLOGISTIC procedure to analyze the binary outcome of folic acid supplementation during pregnancy among Guatemalan women, specifically investigating the interaction between the quality of antenatal care and ethnicity while adjusting to the covariates. We also utilized the GLIMMIX technique to account for the hierarchical nature of our data. By employing a multilevel logistic regression, we examined the influence of individual-, household-, and community-level factors on the quality of antenatal care on folic acid supplementation during pregnancy. The dependent variable is folic acid supplementation with a logistic link function. The default variance function and maximum likelihood estimation were employed to achieve precise parameter estimates. Weights and cluster-level adjustments were applied to account for hierarchical data structure and intracluster similarities. The Gauss–Hermite quadrature was employed to improve the accuracy of the estimates involving random effects, while the Dual Quasi-Newton approach effectively handled the complexity of models with multiple parameters [23].

Community-level variables were derived from individual-level binary variables, including the educational level, media access, and household wealth. First, we created binary indicators for each of these variables at the individual level: education was defined as 0 for no education or primary education and 1 for secondary or higher education; mass media access was defined as 0 for no access to mass media and 1 for access to at least one source of mass media; and wealth was defined as 0 for the poorest and poorer categories and 1 for middle, richer, and richest categories. These binary variables were then aggregated at the community level (cluster) by calculating the proportion of individuals in each cluster who fell into the higher category for each variable. These community-level proportions were subsequently divided into tertiles. These tertiles were included in Model 2 to assess the impact of community-level socioeconomic factors on folic acid supplementation, independent of individual-level characteristics. This approach was chosen for its interpretability and consistency with previous studies utilizing DHS data. The binary classification of wealth allows for clear distinctions between lower and higher economic groups, facilitating meaningful comparisons of socioeconomic disparities across communities. In addition, aggregating these individual-level measures at the cluster level captures broader community characteristics that influence maternal health behaviors.

The intraclass correlation coefficient (ICC) was calculated to quantify the variance in folic acid supplementation due to community differences. A random intercept for community clustering, represented by the variable v021, was incorporated into each model to capture this variability. The modeling process was initiated with a null model, which included only the community-level random intercept without any fixed predictors, serving as a baseline. Model 1 incorporated individual and household-level factors such as demographic and socioeconomic characteristics. Model 2 expanded the analysis to include community-level variables, exploring the impact of broader environmental and accessibility influences. Model 3 included all variables from the previous models, providing a comprehensive analysis of the nested data structure. Model goodness of fit was evaluated using Akaike's Information Criterion (AIC) and Bayesian Information Criterion (BIC), with lower scores indicating a better fit. The odds ratios (ORs) and 95% confidence intervals (CIs) derived from the fixed effects in Model 3 offered a detailed summary of the relationship between antenatal care quality and folic acid supplementation, accounting for various factors across different levels of influence.

The method section follows Preferred Reporting Items for Complex Sample Survey Analysis (PRICSSA) [24]. All analyses were performed using SAS 9.4 (SAS Institute, Cary, NC) [25], accounting for a random intercept for each cluster in a multilevel model.

## 3. Results

### 3.1. Sociodemographic Characteristics

The characteristics of the participants in relation to folic acid supplementation are presented in Table 1. Among the 9523 participants, 42.7% identified as indigenous and 11.9% received adequate antenatal care. In terms of socioeconomic characteristics, 45.5% of the participants were classified as poor, while 34.3% were categorized as the wealthiest. The study population was predominantly rural (62.0%), with 38.0% residing in urban areas. Access to mass media varied; 48.8% of the participants reported exposure to three or more sources, while 8.1% had no access to mass media. Approximately 16.6% of women reported that their last pregnancy was unwanted.

In bivariate analysis, nonsupplementation rates varied by age group. Women aged 35–49 had a higher nonsupplementation rate (18.4%) compared with 13.6% among women aged 25–29 (*p* < 0.001). Education level was also a significant factor (*p* < 0.001); 28.7% of women with no education did not take folic acid, compared with 6.1% among those with secondary or higher education. Marital status showed a notable difference in folic acid supplementation. About 22.2% of never-married women did not take folic acid compared to 14.8% married or living together (*p* < 0.001). Indigenous women had lower folic acid supplementation compared with nonindigenous women (80.7% vs. 88.5%, *p* < 0.001); 21.8% of women from poor households did not take folic acid, in contrast to only 7.3% from rich households (*p* < 0.001). Employment status was associated with folic acid supplementation, with 16.4% of unemployed women not taking folic acid compared with 13.4% of employed women (*p*=0.002). Perceived distance to healthcare facilities was significantly associated with folic acid supplementation, with 19.4% of women citing distance as a big problem, compared with 12.4% who reported distance to healthcare facility, not a big problem (*p* < 0.001). Rural residents also showed lower supplementation, with 17.6% not taking folic acid compared with 11.8% in urban areas (*p* < 0.001).

The total number of children a woman had was negatively associated with folic acid supplementation. Approximately, 24.4% of women with five or more children did not take folic acid, compared with 12.3% of those with only one child (*p* < 0.001). Regarding pregnancy intention, 20.2% of women who did not want more children did not take folic acid, compared with 14.0% who wanted the pregnancy at the time (*p* < 0.001). Lack of mass media exposure was also associated with nonsupplementation; 30.6% of women without access to any media source did not take folic acid supplements, significantly higher than the 8.7% with access to three media sources (*p* < 0.001).

At the community level, folic acid supplementation varied by community-level education, media exposure, and wealth index. Folic acid supplementation decreases as the proportion of individuals with no or primary education increases in the community. In communities where the proportion of individuals with no or primary education was low, 90.7% reported folic acid supplementation, compared with 76.8% in communities with a high proportion of individuals with no or primary education (*p* < 0.001). Similarly, folic acid supplementation was lower in communities with a higher proportion of individuals lacking mass media exposure. Among communities with a low level of individuals lacking mass media exposure, 89.6% reported taking folic acid, compared with 77.5% in communities with a high level of individuals lacking mass media exposure (*p* < 0.001). Folic acid is also higher in communities with lower poverty levels. Among communities with a low proportion of individuals categorized as poor, 90.9% reported folic acid supplementation, compared with 78.0% in communities with a high poverty rate (*p* < 0.001).

### 3.2. Multilevel Modeling

The multilevel logistic regression model in Table 2 examines the factors associated with folic acid supplementation, considering individual and community-level influences.

In Model I, individual and household-level factors were included. Maternal age, marital status, and ethnicity were significantly associated with folic acid supplementation. Younger mothers, particularly those aged 15–19, had lower odds of taking folic acid than older mothers (AOR = 0.60; 95% CI: 0.44–0.81). Similarly, mothers who were never married had significantly lower odds of taking folic acid than those who were married or living together (AOR = 0.53; 95% CI: 0.40–0.70). Indigenous mothers also had reduced odds of folic acid supplementation compared to nonindigenous mothers (AOR = 0.61; 95% CI: 0.53–0.71). The quality of antenatal care was a strong predictor of folic acid supplementation, with mothers receiving no antenatal care having significantly lower odds of folic acid supplementation (AOR = 0.03; 95% CI: 0.02–0.04), and those with intermediate care also had reduced odds compared with mothers with adequate antenatal care (AOR = 0.58; 95% CI: 0.46–0.73).

In Model II, communities with the highest proportion of individuals characterized by low literacy had significantly lower odds of folic acid supplementation (AOR = 0.50; 95% CI: 0.37–0.68) compared with communities with the lowest proportion of low literacy. Similarly, residing in communities with a higher proportion of individuals lacking mass media exposure was associated with reduced odds of folic acid supplementation (AOR = 0.63; 95% CI: 0.50–0.79) compared with communities with a lower proportion of individuals lacking mass media exposure. The wealth index also played a role, as individuals from communities with a higher proportion of poverty had lower odds of folic acid supplementation (AOR = 0.73; 95% CI: 0.54–1.00) compared with communities with a lower proportion of poverty.

In Model III, the results for community-level factors show significant associations with folic acid supplementation, indicating that higher community education levels, media exposure, and wealth are positively associated with folic acid supplementation. Specifically, in communities with a higher proportion of individuals having no or primary education, folic acid supplementation was significantly lower compared to communities with a lower proportion of individuals having no or primary education (AOR = 0.53; 95% CI: 0.38–0.72). In communities with a high percentage of individuals lacking mass media exposure, women have 33% lower odds of taking folic acid compared to those residing in communities with higher mass media access (AOR = 0.67; 95% CI: 0.53–0.84). In communities with a high percentage of poverty, women have 29% lower odds of taking folic acid supplementation compared with those in communities with a low percentage of poverty (AOR = 0.71; 95% CI: 0.51–0.99). The null model, which includes only the random effects at the community level, shows that 16.19% of the variance in folic acid supplementation is attributable to differences between communities. The community variance decreases as more individual and community-level predictors are added, with the final model (Model III) accounting for only 9.09% of the variance at the community level, indicating that the included predictors explain a substantial portion of the differences between communities.

## 4. Discussion

The findings of this study indicate a significant association between the quality of antenatal care and folic acid supplementation in Guatemalan women during pregnancy. The observed association is independent of potential confounders. During antenatal care, healthcare providers must discuss folic acid's benefits for pregnant women and how it can help prevent neural tube defects [26–29]. Antenatal care affords regular health check-ups, maternal and fetal wellbeing monitoring, nutritional guidance, essential supplements, and health behavior education [2]. Effective antenatal care programs, such as the study conducted in Kenya on the effect of community-based health education, can empower women by increasing their knowledge and understanding of the importance of folic acid supplementation during pregnancy and their attitude toward iron and folic acid supplementation.

Sociodemographic factors such as having secondary education or higher, being married, belonging to a rich household, and accessing mass media information were associated with folic acid supplementation among pregnant women. These factors demonstrate the significance of social and economic factors in affecting health behaviors. These findings are supported by several studies demonstrating that socioeconomic status, education, physical environment, employment, and social support networks, among others, positively impact health behaviors and associated health outcomes [30–32].

Our results indicate that rural residents had lower folic acid supplementation compared to urban residents (17.6% vs. 11.8%, *p* < 0.001), which aligns with existing research suggesting that urban areas benefit from better healthcare infrastructure, easier access to healthcare providers, and greater availability of health-related information [33]. Women in rural areas who take folic acid may have access to specific maternal health programs or community-based interventions designed to enhance prenatal nutrition in underserved regions. In addition, family and community support systems in rural settings may play a compensatory role in encouraging folic acid use, even in the presence of healthcare access barriers [34, 35].

Our study supports the WHO's antenatal care recommendations [2]. These guidelines stress the importance of nutritional treatments and education during antenatal care, promoting the need for a minimum of four antenatal contacts to guarantee adequate prenatal support. Moreover, the implications of our research extend into global health policy, particularly supporting the objectives outlined in the Sustainable Development Goals 2030 (SDGs) [36]. Our study contributes practical insights toward achieving Goal 3, which focuses on ensuring healthy lives and promoting wellbeing at all ages. Specifically, our findings reinforce the efforts under Target 3.1, which aims to reduce the global maternal mortality ratio, and Target 3.2, which seeks to end preventable deaths of newborns and children under 5 years of age. Furthermore, the disparities through our research underscore the urgent need to address SDG Target 10, which advocates for reduced inequalities. The identified social determinants of health—education, income, and media access—are crucial areas requiring significant attention to bridge the gaps in maternal healthcare.

Considering Guatemala's unique healthcare landscape, our findings emphasize the necessity for targeted policy interventions to enhance folic acid supplementation. One effective strategy would be integrating nutrition-focused counseling into routine antenatal care visits, ensuring that pregnant women receive consistent information about folic acid's importance. In addition, community-based interventions should prioritize indigenous and low-education populations, using culturally appropriate health education strategies, including peer-led programs and community health worker outreach. To improve access, mass media campaigns such as radio and social media outreach in indigenous languages could raise awareness of folic acid supplementation. Financial incentives, such as conditional cash transfers or transportation subsidies, could also increase antenatal visit attendance, ensuring more women receive essential prenatal supplements. In addition, our findings could inform regional efforts across Central and Latin America, where similar disparities in maternal nutrition and healthcare access persist. Strengthening cross-country collaboration could help harmonize strategies and improve outcomes across the region. Given the observed importance of media exposure, future interventions could also incorporate digital health and mobile-based outreach strategies to promote awareness and uptake of folic acid supplementation, especially in remote and underserved areas.

However, the findings also explain a broader challenge prevalent across Latin America and the Caribbean. With the most recent DHS data for countries in this region ranging from as early as 1987 in Ecuador to as recent as 2017-2018 in Haiti, there is a notable disparity in the availability of current health data. For instance, the latest data for Bolivia is from 2008, Colombia from 2015, and Guatemala, from which this study draws from 2014 to 2015 [37]. This inconsistency significantly impedes a comprehensive understanding of the region's public health needs and the development of timely interventions. These countries may have been gathering their data. However, the efforts of organizations like DHS to consolidate and widely distribute information have resulted in a uniform and globally comparable measure of health indicators.

While we included media access as a variable, thereby considering that health promotion education can be disseminated through media outlets, this dataset lacked information on social media access, which may be more relevant to women of childbearing age, as suggested by the recent online campaign to promote antenatal care in Guatemala [38]. The study did not include data on women's perception of folic acid and availability (including supply chain issues). In addition, the cross-sectional nature of the data limits our ability to establish causality between the quality of antenatal care and folic acid supplementation. Thus, the observed associations should be interpreted as correlational rather than causal. There is also a potential for bias introduced by the reliance on self-reported data, which can lead to recall inaccuracies or social desirability bias, potentially skewing the reported behaviors. These factors should be carefully considered when interpreting the results and planning future research directions.

In conclusion, we found an association between the adequate quality of antenatal care and folic acid supplementation among pregnant women in Guatemala. This supports the role of quality and comprehensive antenatal care services in promoting essential supplements such as folic acid, thus contributing to maternal and child health that aligns with the WHO recommendations. To improve folic acid supplementation, national policies should support the fortification of staple foods, such as maize flour, and integrate stronger nutrition counseling into prenatal care visits. Ensuring that healthcare providers routinely discuss folic acid supplementation can help increase awareness and adherence, especially in underserved communities.

Beyond Guatemala, these findings emphasize a broader need for stronger maternal health policies in similar low-resource settings. Governments and health agencies should prioritize improving access to antenatal care, expanding community health education, and increasing the availability of supplements. Furthermore, better coordination among countries in the region could enhance data collection and standardize maternal health programs, ensuring that resources are used effectively to support women and newborns.

## Figures and Tables

**Table 1:** Characteristics of the study sample by folic acid intake (*N* = 9523).

	Overall *n* (wt. %)	Folic acid		*p*
		No *n* (wt. %)	Yes *n* (wt. %)	
Individual and household-level factors				
Maternal age				< 0.001
15–19	934 (9.6)	162 (18.3)	772 (81.7)	
20–24	2524 (26.8)	365 (14.4)	2159 (85.6)	
25–29	2426 (25.5)	305 (13.6)	2121 (86.4)	
30–34	1859 (19.9)	260 (14.9)	1599 (85.1)	
35–49	1780 (18.2)	327 (18.4)	1453 (81.6)	
Maternal education				< 0.001
None	1598 (17.2)	447 (28.7)	1151 (71.3)	
Primary	4937 (51.4)	786 (16.6)	4151 (83.4)	
Secondary or above	2988 (31.4)	186 (6.1)	2802 (93.9)	
Marital status				< 0.001
Never married	509 (5.3)	110 (22.2)	399 (77.8)	
Married/living together	8155 (86.0)	1160 (14.8)	6995 (85.2)	
Divorced/widowed/separated	859 (8.7)	149 (16.8)	710 (83.2)	
Ethnicity by auto identification^∗^				< 0.001
Not indigenous	5069 (52.3)	590 (11.5)	4479 (88.5)	
Indigenous	4013 (42.7)	827 (19.3)	3625 (80.7)	
Household wealth index				< 0.001
Poor	4438 (45.5)	913 (21.8)	3525 (78.2)	
Middle	1953 (20.2)	279 (14.6)	1674 (85.4)	
Rich	3132 (34.3)	227 (7.3)	2905 (92.7)	
Maternal employment				0.002
Not employed	6339 (66.6)	1011 (16.4)	5328 (83.6)	
Employed	3184 (33.4)	408 (13.4)	2776 (86.6)	
Distance				< 0.001
No big problem	5509 (57.3)	671 (12.4)	4838 (87.6)	
Big problem	4014 (42.7)	748 (19.4)	3266 (80.6)	
Place of residence				< 0.001
Urban	3479 (38.0)	396 (11.8)	3083 (88.2)	
Rural	6044 (62.0)	1023 (17.6)	5021 (82.4)	
Number of children born				< 0.001
1 child	2932 (30.3)	346 (12.3)	2586 (87.7)	
2–4 children	5431 (57.7)	796 (15.2)	4635 (84.8)	
5 or more	1160 (12.0)	277 (24.4)	883 (75.6)	
Pregnancy intention				< 0.001
Wanted then	5941 (62.2)	780 (14.0)	5161 (86.0)	
Wanted later	2016 (21.2)	321 (15.7)	1695 (84.3)	
Wanted no more	1566 (16.6)	318 (20.2)	1248 (79.8)	
Mass media access				< 0.001
None	784 (8.1)	234 (30.6)	550 (69.4)	
1 source	1530 (15.5)	348 (24.4)	1182 (75.6)	
2 sources	2602 (27.6)	441 (17.5)	2161 (82.5)	
3 sources	4607 (48.8)	396 (8.7)	4211 (91.3)	
Quality of antenatal care				< 0.001
None	352 (3.8)	260 (74.5)	92 (25.5)	
Intermediate	8117 (84.3)	1063 (13.7)	7054 (86.3)	
Adequate	1054 (11.9)	96 (8.8)	958 (91.2)	
Folic acid supplementation				
No	1419 (15.4)	—	—	
Yes	8104 (84.6)	—	—	
Community-level factors				
% No and primary education				< 0.001
Low	3447 (36.2)	304 (9.3)	3143 (90.7)	
Medium	3177 (33.4)	441 (13.4)	2736 (86.6)	
High	2899 (30.4)	672 (23.2)	2227 (76.8)	
% No media exposure				< 0.001
Low	5179 (54.4)	535 (10.4)	4641 (89.6)	
Medium	1414 (14.8)	223 (16.3)	1191 (83.7)	
High	2933 (30.8)	659 (22.5)	2275 (77.5)	
% Poor wealth index				< 0.001
Low	3467 (36.4)	313 (9.1)	3154 (90.9)	
Medium	3203 (33.6)	475 (15.5)	2728 (84.5)	
High	2853 (30.0)	629 (22.0)	2225 (78.0)	

*Note:* Wt.%: weighted percent. Rao–Scott chi-square *p* values.

^∗^ *N* = 9521.

**Table 2:** Multilevel logistic regression analysis of factors on folic acid supplementation.

	Null model OR (95% CI)	Model I AOR (95% CI)	Model II AOR (95% CI)	Model III AOR (95% CI)
Individual and household-level factors				
Maternal age				
15–19	—	0.60 (0.44–0.81)	—	0.67 (0.49–0.90)
20–24	—	0.86 (0.68–1.08)	—	0.91 (0.72–1.16)
25–29	—	1.03 (0.83–1.30)	—	1.08 (0.86–1.35)
30–34	—	1.00 (0.81–1.24)	—	1.03 (0.83–1.27)
35–49	—	1	—	1
Marital status				
Never married	—	0.53 (0.40–0.70)	—	0.53 (0.40–0.70)
Married/living together	—	1	—	1
Divorced/widowed/separated	—	0.80 (0.64–1.01)	—	0.78 (0.62–0.99)
Ethnicity by auto identification				
Not indigenous	—	1	—	1
Indigenous	—	0.61 (0.53–0.71)	—	0.74 (0.63–0.86)
Maternal employment				
Not employed	—	0.88 (0.76–1.02)	—	0.94 (0.81–1.09)
Employed	—	1	—	1
Pregnancy intention				
Wanted then	—	1.15 (0.97–1.37)	—	1.15 (0.97–1.37)
Wanted later	—	1.08 (0.88–1.33)	—	1.06 (0.86–1.30)
Wanted no more	—	1	—	1
Number of children born				
1 child	—	1	—	1
2–4 children	—	0.68 (0.57–0.82)	—	0.72 (0.60–0.87)
5 or more	—	0.45 (0.34–0.59)	—	0.53 (0.40–0.70)
Quality of antenatal care				
None	—	0.03 (0.02–0.04)		0.03 (0.02–0.04)
Intermediate	—	0.58 (0.46–0.73)		0.59 (0.47–0.74)
Adequate	—	1		1
Distance				
No big problem	—	1		1
Big problem	—	0.80 (0.70–0.91)		0.86 (0.75–0.98)
Residencia				
Rural		0.75 (0.62–0.90)		1.31 (1.05–1.64)
Urban		1		1
Community-level factors				
% No and primary education				
Low	—	—	1	1
Medium	—	—	0.80 (0.63–1.03)	0.83 (0.64–1.07)
High			0.50 (0.37–0.68)	0.53 (0.38–0.72)
% No media exposure				
Low	—	—	1	1
Medium	—	—	0.79 (0.62–1.01)	0.81 (0.63–1.04)
High			0.63 (0.50–0.79)	0.67 (0.53–0.84)
% Poor wealth index				
Low	—	—	1	1
Medium	—	—	0.81 (0.63–1.04)	0.74 (0.57–0.97)
High			0.73 (0.54–1.00)	0.71 (0.51–0.99)
Random effects				
Community variance	0.66 (0.07)	0.45 (0.06)	0.39 (0.06)	0.33 (0.05)
ICC%	16.19%	12.09%	10.58%	9.09%
Model comparison				
AIC	7893.79	7125.82	7739.22	7047.92
BIC	7903.29	7211.36	7777.24	7161.97
Fit statistics for conditional distribution				
−2 log L(folic acid |*r*. effects)	7117.38	6522.30	7176.28	6552.01
Pearson chi-square	7940.90	8154.03	8308.06	8323.69
Pearson chi-square/DF	0.83	0.86	0.87	0.87

Abbreviation: DF = degrees of freedom.

## Data Availability

The data that support the findings of this study are available from the DHS Program (https://dhsprogram.com). Restrictions apply to the availability of these data, which were used under license for this study. Data are available from the DHS Program upon request and with permission.
